# Autoantibodies in a Three-Year-Old Girl with Visceral Leishmaniasis: A Potential Diagnostic Pitfall

**DOI:** 10.1155/2016/2081616

**Published:** 2016-06-23

**Authors:** Gholamreza Pouladfar, Zahra Jafarpour, Amir Hossein Babaei, Bahman Pourabbas, Bita Geramizadeh, Anahita Sanaei Dashti

**Affiliations:** ^1^Professor Alborzi Clinical Microbiology Research Center, Namazi Hospital, Shiraz University of Medical Sciences, Shiraz, Iran; ^2^Student Research Committee, Shiraz University of Medical Sciences, Neshat Street, Shiraz 71348 43638, Iran; ^3^Transplant Research Center, Pathology Department, Shiraz University of Medical Sciences, Shiraz, Iran

## Abstract

Visceral leishmaniasis (VL), a life-threatening parasitic infection, is endemic in the Mediterranean region. Diagnosis of VL is based on epidemiologic, clinical, and laboratory findings. However, sometimes, clinical features and laboratory findings overlap with those of autoimmune diseases. In some cases, autoantibodies are detected in patients with VL and this could be a potential diagnostic pitfall. In this study, we have reported on a three-year-old girl from a VL-endemic area in Iran, who presented with prolonged fever and splenomegaly. Bone marrow examination, serologic tests, and the molecular PCR assay were performed; however, results were inconclusive. The levels of anti-double stranded DNA, cytoplasmic antineutrophil cytoplasmic autoantibody, and perinuclear antineutrophil cytoplasmic autoantibody were elevated and, at the end, splenic biopsy was performed. The splenic tissue PCR test detected the DNA of* Leishmania infantum*. The patient's condition improved with anti-*Leishmania* therapy, and the autoantibodies disappeared within the following four months. Clinical presentations and laboratory findings of VL and autoimmune diseases may overlap in some patients.

## 1. Introduction

Visceral leishmaniasis (VL) is an endemic parasitic infection, occurring in India, Africa, South America, the Middle East region, and the eastern Mediterranean region. VL is an endemic disease in northwest (Ardebil and East Azerbaijan provinces) and southwest (Fars and Bushehr provinces) of Iran [1]. It could be life-threatening if diagnosis is delayed and treatment is inappropriate [2]. In the eastern Mediterranean region,* Leishmania infantum* (*L. infantum*) is the main cause of VL. In Iran,* L. infantum* is the leading cause of VL, while* L. tropica* has been recognized as the second most common cause [3, 4].

Suspicion of VL is usually based on demographic, clinical, and laboratory findings. A patient with VL shows a number of clinical features such as fever, malaise, weight loss, and hepatosplenomegaly, as well as some nonspecific laboratory findings like pancytopenia, hypergammaglobulinemia, elevated C-reactive protein (CRP) level, and high erythrocyte sedimentation rate (ESR). These clinical features and laboratory findings could mimic those of autoimmune diseases [5, 6]. Sometimes, autoantibodies such as antinuclear antibody (ANA), anti-double stranded DNA (anti-dsDNA), cytoplasmic antineutrophil cytoplasmic autoantibody (C-ANCA), perinuclear antineutrophil cytoplasmic autoantibody (P-ANCA), rheumatoid factor (RF), and anti-smooth muscle antibodies (ASMA) are detected in patients with VL. These may lead to a potential diagnostic pitfall [5, 7]. Indeed, several reports have described the misdiagnosis of VL as an autoimmune disease which has led to fatal outcomes [7, 8].

In this work, we report the case study of a three-year-old girl with the final diagnosis of visceral leishmaniasis, where autoimmune antibodies in the serum were elevated, resulting in an initial misleading diagnosis.

## 2. The Case

A three-year-old girl was referred to the Namazi Teaching Hospital, affiliated to Shiraz University of Medical Sciences, with a history of fever for seven days, as well as splenomegaly. She was referred from an endemic area of VL in Fars province, southeastern Iran. The notable findings in the general physical examination were an axillary temperature of 40°C and a palpable spleen just below the costal margin. Based on the history and physical examination there were no other explanations for other sources of infection.

On admission, a complete blood count revealed pancytopenia. The hemoglobin level was 9.3 g/dL, and there were a mean corpuscular volume of 77 femtoliters, white blood cell (WBC) count of 4400/mm^3^ with 35% neutrophils, 62% lymphocytes, and a platelet count of 119000/mm^3^. The reticulocyte count was 1.2% and the direct and indirect Coombs tests were negative. Liver function test results were as follows: albumin 3.8 mg/dL, globulin 2.8 mg/dL, alanine aminotransferase (ALT) 16 U/L, aspartate aminotransferase (AST) 51 U/L, alkaline phosphatase 267 U/L, serum lactate dehydrogenase 1474 U/L (normal level below 480 U/L), total bilirubin 0.5 mg/dL, and direct bilirubin 0.1. Prothrombin time and partial thromboplastin time were within the normal range. The systemic inflammation indices were abnormal, including an ESR of 75 mm/h and a CRP level of 11 mg/dL. The ferritin level was 690 ng/mL (normal: 10–124 ng/mL). The renal function was normal.

Urine, blood, and stool cultures were also negative. In thick and thin smears for malaria, no parasites were detected. The DNA quantitative PCR for cytomegalovirus and EBV, the Wright and Widal tests, and HIV antibody were all negative. The Epstein-Barr virus, viral-capsid antigen (EBV-VCA) IgM was within the normal range. The Venereal Disease Research Laboratory (VDRL) test for syphilis and the cold agglutinin tests were also negative. The serum immunofluorescence assay (IFA) for determining* Leishmania* antibodies and quantitative PCR for detecting* L. infantum* kinetoplast DNA were performed by methods described previously and results were negative [4, 9].

Some autoimmune lab findings, including RF, ANA, anticardiolipin antibodies (ACLA), anti-Smith (anti-Sm), anti-Sjögren's syndrome related antigen A (anti-SSA/Ro), anti-Sjögren's syndrome related antigen B (anti-SSB/La), and anti-topoisomerase I (anti-Scl-70) antibodies, were negative. On the other hand, others were positive including anti-dsDNA, 42.2 U/mL (positive above 24), C-ANCA, 27.3 U/mL (positive above 18), and P-ANCA, 26.3 U/mL (positive above 18). The serums C3 and C4 were within normal range.

Abdominal ultrasonography and CT scan both confirmed splenomegaly. Bone marrow smears and biopsy indicated mild hypocellular marrow; however the myeloid and erythroid maturation was normal. The PCR test for detection of* L. infantum* in bone marrow was negative. There was no evidence of malignancy or an infiltrative disease and no Leishman bodies were seen.

For evaluating the competency of the immune system, quantitative serum immunoglobulin tests, the CH50 assay, the dihydrorhodamine (DHR) flow cytometric test, and the T-cell and B-cell counts by flow cytometry were performed, and their results were normal.

One week after admission, the patient's condition started to deteriorate and pancytopenia worsened (hemoglobin 6.1 mg/dL, WBC 2.8/mm^3^, and platelet 29000/mm^3^). Despite the negative results in our initial investigations for VL diagnosis, we made the decision to start VL treatment with amphotericin B deoxycholate (AmB-d, 1 mg/kg per day by infusion, daily). This decision was made based on demographic data, clinical and laboratory findings, and exclusion of other probable causes.

**Figure 1:**
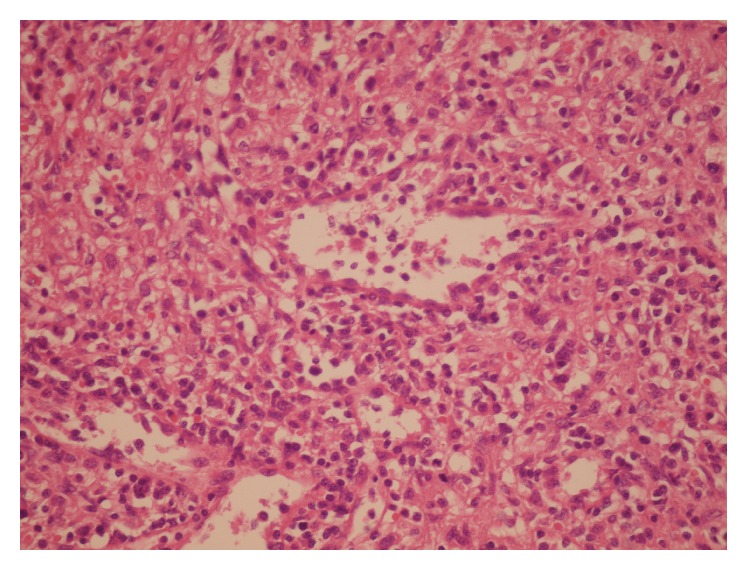
Splenic section H&E; red pulps expanded by heavy infiltration of plasma cells and histiocytes. No Leishman body is present.

One week after AmB-d administration, the patient's condition started to improve dramatically. Ultimately, VL was diagnosed when an open spleen biopsy was performed. A histological examination of the spleen biopsy showed red pulp expansion with heavy infiltration of plasma cells and histiocytes. This was highly suggestive of VL. No Leishman bodies were detected (Figure 1). The qualitative PCR in the spleen tissue revealed* L. infantum* kinetoplast DNA. On the tenth day of treatment with AmB-d, the platelet count started to increase; therefore, we changed AmB-d to intramuscular Glucantime at 20 mg/kg per day for a period of 20 days.

Four months after her therapy, the patient was in good clinical condition and free of any complaints. She had normal blood counts, inflammatory indices, and biochemical analyses. Anti-dsDNA, C-ANCA, and P-ANCA levels had become normal (3.24 U/mL, 2.14 U/mL, and 1.42 U/mL, resp., normal below 5 U/mL).

## 3. Discussion

Clinical presentations and laboratory findings of VL and autoimmune diseases may overlap in some cases. The similarity in clinical presentations (fever, pallor, anorexia, malaise, weight loss, and hepatosplenomegaly) and laboratory findings (anemia, leucopenia, thrombocytopenia, hypergammaglobulinemia, hypoalbuminemia, low serum complement levels, high levels of inflammatory markers like ESR and CRP, and the presence of anti-dsDNA, C-ANCA, and P-ANCA) could therefore be misleading in differentiating VL from autoimmune diseases [10, 11].

It was previously reported that some patients with VL had been misdiagnosed with autoimmune hepatitis [12, 13], primary biliary cirrhosis, and systemic lupus erythematosus [7, 14]. These misdiagnoses sometimes led to fatal outcomes [7, 8]. In addition, VL could develop in patients with autoimmune diseases who were treated with immunosuppressive medications. Overlapping clinical manifestations could lead to delayed diagnosis of VL and fatal consequences [15, 16].

Sometimes autoantibodies like ANA, P-ANCA, C-ANCA, RF, anti-dsDNA, anti-Sm, anti-SSA/Ro, anti-SSB/La, and ASMA are detected in patients with VL [5, 7]. There are three major theories for the formation of autoantibodies in VL patients. The first theory claims that the destruction of tissues by the protozoa causes the release of self-antigens and their exposure to the immune system. The second theory claims that polyclonal B-cell activation and the altered function of regulatory and suppressor T-cells lead to the formation of autoantibodies. The last theory claims that molecular mimicry of the host antigens by antigens of* Leishmania* can cause a cross-reactivity [5, 7, 13].

The gold standard of VL diagnosis still remains the detection of parasites in the bone marrow aspiration or splenic tissue by smear or culture [17]. Indeed, splenic smears have the highest sensitivity for detecting VL (93.1–98.7%), compared to bone marrow and lymph node smears (52–85% and 52–58%, resp.). Serological tests such as IFA, which are frequently performed in areas where VL is endemic, serve as a highly sensitive diagnostic method in immunocompetent patients. The sensitivity and specificity of IFA for VL are shown to be 96% and 98%, respectively [18]. Molecular tests such as PCR have been proposed as highly sensitive methods in VL diagnosis. In a recent meta-analysis, the pooled sensitivity of PCR in whole blood was 93.1% (95% confidence interval (CI), 90.0 to 95.2), and the specificity was 95.6% (95% CI, 87.0 to 98.6) [17]. The sensitivity of the PCR assay in bone marrow aspirate samples is 95.7% [19]. There have not been many studies investigating the sensitivity and specificity of PCR in human spleen tissues. In a study carried out in southern Iran, PCR revealed the existence of parasite DNA in all 22 splenic aspirate specimens of confirmed VL patients [9]. In the patient of this study, splenic tissue PCR was the only diagnostic test that confirmed VL.

In conclusion, the presence of autoantibodies can be a potential pitfall which can lead to the diagnosis of autoimmune diseases instead of VL. Maintaining high clinical indexes for suspicion of VL in patients who are referred from endemic areas plays a crucial role in efficiently managing and treating patients, unless clinical findings and lab tests rule out VL suspicion.
